# Towards Rational Biosurfactant Design—Predicting Solubilization in Rhamnolipid Solutions

**DOI:** 10.3390/molecules26030534

**Published:** 2021-01-20

**Authors:** Ilona E. Kłosowska-Chomiczewska, Adrianna Kotewicz-Siudowska, Wojciech Artichowicz, Adam Macierzanka, Agnieszka Głowacz-Różyńska, Patrycja Szumała, Krystyna Mędrzycka, Elżbieta Hallmann, Elena Karpenko, Christian Jungnickel

**Affiliations:** 1 Department of Colloid and Lipid Science, Faculty of Chemistry, Gdańsk University of Technology, Narutowicza St. 11/12, 80-233 Gdańsk, Poland; adriannakotewicz@gmail.com (A.K.-S.); adamacie@pg.edu.pl (A.M.); agnglowa@pg.gda.pl (A.G.-R.); patszuma@pg.edu.pl (P.S.); kbm@pg.edu.pl (K.M.); hallmann.elzbieta@gmail.com (E.H.); cjungnickel@googlemail.com (C.J.); 2 Department of Hydraulic Engineering, Faculty of Civil and Environmental Engineering, Gdańsk University of Technology, Narutowicza St. 11/12, 80-233 Gdańsk, Poland; wojartic@pg.edu.pl; 3 Department of Physical Chemistry of Fossil Fuels InPOCC, National Academy of Sciences of Ukraine, 3a Naukova St., 79053 Lviv, Ukraine; e.v.karpenko@gmail.com

**Keywords:** rhamnolipid, biosurfactant, micellar solubilization, efficiency, MSR, QSAR, prediction

## Abstract

The efficiency of micellar solubilization is dictated inter alia by the properties of the solubilizate, the type of surfactant, and environmental conditions of the process. We, therefore, hypothesized that using the descriptors of the aforementioned features we can predict the solubilization efficiency, expressed as molar solubilization ratio (MSR). In other words, we aimed at creating a model to find the optimal surfactant and environmental conditions in order to solubilize the substance of interest (oil, drug, etc.). We focused specifically on the solubilization in biosurfactant solutions. We collected data from literature covering the last 38 years and supplemented them with our experimental data for different biosurfactant preparations. Evolutionary algorithm (EA) and kernel support vector machines (KSVM) were used to create predictive relationships. The descriptors of biosurfactant (logP_BS_, measure of purity), solubilizate (logP_sol_, molecular volume), and descriptors of conditions of the measurement (T and pH) were used for modelling. We have shown that the MSR can be successfully predicted using EAs, with a mean R^2^_val_ of 0.773 ± 0.052. The parameters influencing the solubilization efficiency were ranked upon their significance. This represents the first attempt in literature to predict the MSR with the MSR calculator delivered as a result of our research.

## 1. Introduction

Next to wetting, mobilization, and emulsification, solubilization is a common mechanism supporting a variety of domestic and industrial processes, starting from everyday hygiene, washing dishes and doing laundry, including environmental protection and drug delivery systems. For example, in environmental protection, solubilization is an inherent stage of water and ground remediation [1,2,3], which results in a significant increase of pollutants bioavailability for compatible microorganisms [4], therefore supporting self-purification or engineered purification of contaminated sites. In regard to drug delivery systems, solubilization serves not only for the preparation of normally water insoluble drug formulations, but also for their targeted delivery, e.g., in anti-cancer therapies [5,6]. Moreover, solubilization forms an important aspect of the efficient functioning of our digestive system, where human surfactants, i.e., bile salts solubilize consumed fat in order to increase the interfacial surface for enzymatic action of lipase/colipase complex [7].

For any given solubilization application it is crucial to reduce the environmental and human impact, and at the same time provide high efficiency. The human impact of solubilization is especially pronounced by the surfactants’ tendency to irritate human skin in domestic applications, whereas the environmental impact is mostly characterized by their resistance to biodegradation [8,9]. Therefore, an alternative to synthetic surfactants is commonly found among natural surfactants, i.e., biosurfactants, with rhamnolipids (RLs) being the most widely examined and commercially available group [10,11].

RLs are glycolipids produced by variety of microorganisms, often pathogenic, to support foundations of microbial life, i.e., food absorption or biofilm development [12,13]. For industrial purposes however, special emphasis is paid to biosurfactant production by non-pathogenic strains [14,15,16,17]. It allows not only to meet health and safety requirements in bioprocessing, but also to satisfy high requirements for raw materials for pharmaceuticals and cosmetics production. RLs are considered being active in a wide range of pH [18,19] and salinity [19], low- or non-toxic [20], easily biodegradable [20,21], and mild to the skin [22,23]. Except positive effects of RLs, there were reported cases where the biosurfactant either had no impact or even influenced the process negatively. The latter was rather matrix-dependent, i.e., resulted from the presence of co-contaminants next to RLs [24]. Such cases concerned e.g., preferential use of RL as a carbon source by bacteria and resulting lack of stimulation of hydrocarbon degradation [25] or increase of diesel oil phytotoxicity [26]. Notwithstanding, these do not undermine the general overtone of literature, bringing RLs to the position of green surfactants. Not without significance are the facts that CMC of RLs is low compared to synthetic surfactants (Table S1), and they were reported to solubilize hydrocarbons at concentrations even below the CMC [27]. These altogether make RL biosurfactants suitable for many domestic and industrial applications, implying they can be applied at reduced quantities, compared to synthetic surfactants. In spite of vast number of research on efficient methods for RLs production [28,29,30], the overall cost of the synthesis is high, and therefore limits the potential to widely replace surfactants with petrochemical origin. In the scientific world, multidimensional efforts are made to overcome this obstacle [31].

The efficiency of solubilization is most commonly presented in the form of weight or molar solubilization ratio (WSR and MSR, respectively) and is expressed as the ratio of the amount of solubilizate to the amount of surfactant used, as shown in Equation (1):

(1) $$\mathrm{MSR}\;=\;\frac{C-C_{CMC}}{C_{surf}-CMC}$$

where *C* is the concentration of solubilized compound in a micellar solution, *C_CMC_* is the solubility of solubilized compound in a micellar solution at the *CMC*, *C_Surf_* is the concentration of the surfactant, and *CMC* is the critical micellar concentration of the surfactant. For instance, the MSR for *n*-dodecane varied from 0.27 [32] to even 2.91 [27], and the MSR for *n*-hexadecane was 5.2 [33].

The MSR may vary upon different factors connected with the rhamnolipid itself, the solubilizate, but also upon the experimental/industrial conditions. The MSR is crucial for the application of biosurfactants for several purposes in the industry. Being able to predict the MSR, one could more easily find an efficient surfactant for target application and limit the number of experiments needed for the research. In case of rhamnolipids, this could also be a step forward to design biosynthesis of these biomolecules, as the process usually results in obtaining a mixture of different congeners. The congeners differ in basic physicochemical properties, e.g., CMC or the ability to stabilize emulsions. Therefore, a deep understanding of structure–property dependences would allow for the design of the mixtures of rhamnolipids with properties adjusted for different applications. This could be reached by applying quantitative structure–property relationship (QSPR) models. The QSPR is nowadays one of the most commonly used in silico method for testing chemicals [34]. It is a tool for computational modelling, which shows molecular structures of tested chemicals in terms of descriptors later used to predict physical and biological properties [35].

To determine physical or biological activity quantitatively, minimal concentration of tested chemical giving some response is used. By analysis of those responses we can evaluate parameters having the highest influence on the activity of tested compounds. To obtain such information physicochemical parameters (descriptors) are used in QSPR models. Descriptors characterize tested chemical and should be selected by taking into account their relevance for the property under investigation. Different features of the molecule, such as thermodynamic, structural, and electron, can be described by descriptors. QSPR analysis allows then for the determination of most significant descriptors. In addition, it allows for the prediction of the properties of investigated compound within a certain descriptor range.

Even though methods of QSPR for surfactants [36] and ionic liquids are common [37], surprisingly no one has yet attempted to predict the MSR. This is especially unusual, considering the industrial importance of the parameter. Commonly, in technological applications relying on incorporation of insoluble drugs or active anti-aging substances into micelles or other carriers, the general rule is the larger the MSR, the better. However, this is not always the case; for example, a lower MSR opens the possibility to separate components in biological matrices or model non-aqueous phase liquids of environmental importance [38], as shown in Table 1.

Biosurfactants have a wide range of characteristics, with a wide range of MSRs as shown in Table 2. The question is, therefore, which biosurfactant is most suitable for which application? To answer that question, we need to be able to predict the MSR. Even though methods for predicting micelles formation are common [36,44], prediction of the MSR has never been attempted. The only literature example of a related concept concerns microemulsions. The hydrophilic–lipophilic deviation (HLD) concept [45,46] was used to determine the composition of surfactant formulations for cosmetics, drug delivery and detergency [47], oil spills removal [48], enhanced oil recovery [49], also considering biosurfactant application [47]. However, the concept uses several descriptors that have to be determined experimentally prior to calculation, i.e., optimal salinity, critical curvature, equivalent carbon atoms number and empirical constant K. In addition, the HLD concept concerns Winsor type III microemulsion. This middle phase, bicontinuous system is known for its high solubilization capacity, but can be formed only in a narrow salinity [50,51], and temperature range [47]. That excludes many practical applications of the microemulsion, as even simple dilution of the formulation for cleaning purposes would disturb the equilibrium of the system and lead to transformation to Winsor type I or type II.

Therefore, the purpose of this research was to use a series of chemical and phenomenological descriptors to predict the solubilization efficiency of different compounds in biosurfactant solutions, and then rank them upon their significance. The prediction will use the descriptors of both, the biosurfactant, and the solubilizate, but also the conditions of the measurement, which to our knowledge, is a first such attempt. The data used for this come from literature [1,4,27,33,53,54,55,56,57,58,59,60,61,62,63,64,65,66,67,68] as well as experiments.

## 2. Results and Discussion

### 2.1. Solubilizing Properties of Rhamnolipid Biocomplex

For QSPR models it is crucial to have a wide applicability domain, i.e., the space determined by descriptors on which the model was built and where it will be further applicable [69]. The literature dataset covered only narrow purity range of biosurfactants. In order to build a model covering a wider range of biosurfactants we therefore examined a new type of biosurfactant, i.e., rhamnolipid biocomplex with alginate (RBC). RBC belongs to 3rd class of purity according to classification published before [36], that was hardly represented in the literature dataset collected (ESI MSR dataset with MICE). What is more, rhamnolipid biocomplex is a cheaper and more environmentally friendly alternative than purified rhamnolipids [36].

**Figure 1:**
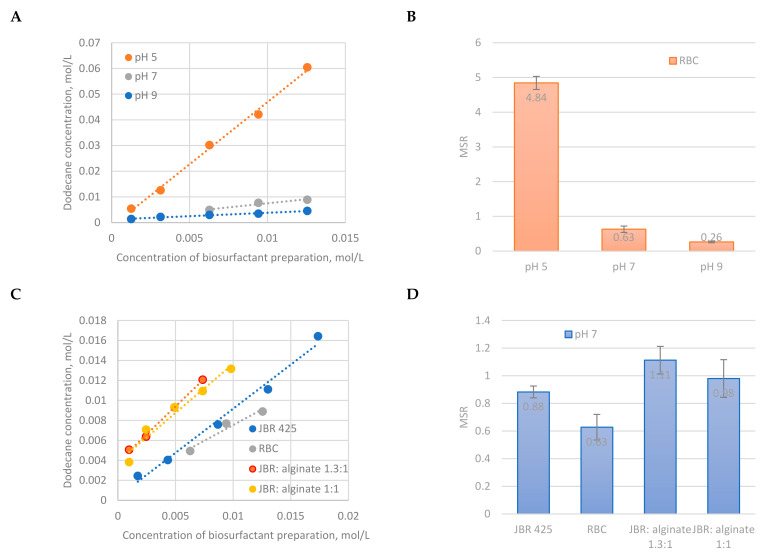
Efficiency of dodecane solubilization in biosurfactant solutions: (**A**) The influence of pH on the dodecane concentration in rhamnolipid biocomplex solutions (RBC) and (**B**) corresponding molar solubilization ratios (MSRs) values; (**C**) dodecane concentration dependence on the concentration of different biosurfactant preparations at pH 7, and (**D**) corresponding MSRs. JBR represents pure RLs (JBR425 of Jeneil), mixtures of JBR and alginate represent model RBC. With increasing pH rhamnolipid aggregates change from vesicles to micelles and the sharp decrease of solubilization efficiency is observed for RBC solutions.

We have determined the influence of pH on the solubilization efficiency of dodecane in RBC solutions (Figure 1A). We observed that the concentration of dodecane increased with increasing RBC concentration. This is due to parallel increase of number of micelles to receive solubilizate. The increase was very steep at pH 5 and significantly milder at pH 7 and 9. The change of slope was obviously reflected by the decrease of MSR values from 4.84 ± 0.19 in acidic to 0.26 ± 0.02 in alkaline conditions (Figure 1B). This can be explained by change of solubility and dissociation of RLs with change of pH. RLs are weak acids with pK_a_ of 5.6 [70]. At pH 5 RL-carboxylic groups are protonated and of limited solubility. We hypothesize that solubilizate may then act as solubility nuclei that supports formation of micelles. Pre-micelles formation was previously described in literature [71], except in our case they would be filled with the solubilizate. It was already reported that RLs are able to solubilize hydrocarbons at concentrations below the CMC [27]. With change of pH, surfactant aggregation behavior changes as well. Shin et al. [72] found that rhamnolipids form vesicles in acidic, and micelles in alkaline conditions. The latter have significantly lower solubilization capacity.

We have also performed solubilization of dodecane in the solutions of different biosurfactant preparations, namely with use of model RBC (i.e., pure RLs mixtures with alginate at different mass ratios) and pure RLs (JBR 425) (Figure 1C). The experiments were performed at pH 7, so that the influence of alginate on solubilization is not overshadowed by the impact of dissociation, solubility or pre-micellization of RLs, as discussed above. Surprisingly, no clear trend was observed. The highest MSR was found for model RBCs, i.e., JBR: Alginate mixtures (1.11 ± 0.10 and 0.98 ± 0.14 for 1.3:1 and 1:1 weight ratio, respectively) as compared to 0.88 ± 0.04 for pure RLs (Figure 1D). Therefore, one could conclude that the presence of alginate increases solubilizing properties or RLs although alginate is not surface active. However, the biocomplex had the lowest MSR (0.63 ± 0.09), although containing about 80% of RLs. It is possibly due to the presence of junk molecules. JBR is composed of pure RLs, and model biocomplexes are composed of JBR and alginate, whereas RBC is a product of biosynthesis that was not so deeply purified. It represents 2nd Impurity class as compared to JBR 425, which represents 0 Impurity class. It was already stated by us previously that junk molecules increase the CMC of RLs. At pH 7 the CMC of JBR was 41.5 mg/L, whereas it was 62.1 mg/L for RBC [36]. Junk molecules may weaken the solubilizing properties of RLs analogously to micellization properties. Increase of MSR observed for model RBCs (Figure 1D) would indicate change of interactions between RLs and alginate with increasing concentration of the latter one. Perhaps some adsorption at alginate interface or even saturation may occur, likewise in surfactant-protein systems [73]. A more detailed physicochemical research would be required to confirm that hypothesis.

The obtained MSR values for RBC, pure RLs, and their mixtures with alginate (model RBCs) at different pH conditions were further used to expand the applicability domain of the model to predict MSR in different biosurfactant systems (ESI MSR dataset).

### 2.2. Predicting MSR with EA and KSVM

**Figure 2:**
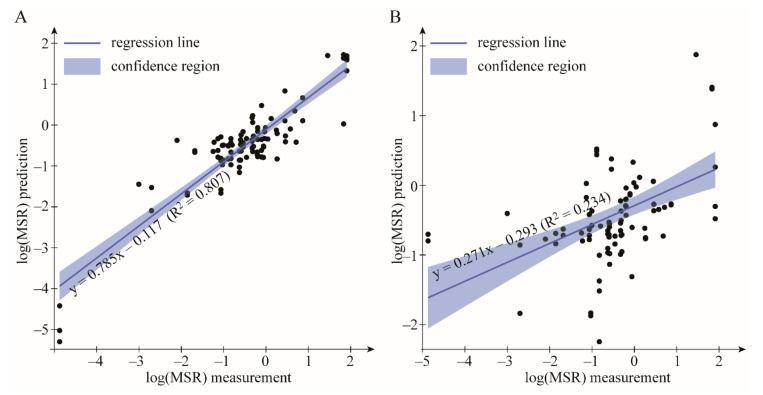
The estimated predictive power of the model used for logMSR calculation. The opaque region is 95% confidence interval (CI) of the regression. As can be seen the evolutionary algorithm (EA) approach performs better (**A**) than the kernel support vector machines (KSVM) (**B**). Variable *x* in regression line equations denotes the log(MSR) measurement. The R^2^ values displayed in the figures are given with respect to outcomes of all ten testing sets.

The model to predict MSR was built on the combined literature and experimental data and represented by the function logMSR = f(CMC, pH, logP_sol_, V_m sol_, T, (im)purity, logP_BS_) with evolutionary algorithm (EA) EureqaPro was generated 10 times. The results of the modelling are presented in Table 3 and Figure 2.

The external validation results showed an advantage of the EA over KSVM model. The average R^2^_val_ for ten validation sets were equal to 0.773 ± 0.052 and 0.166 ± 0.152 for EA and KSVM, respectively (Table 3). The overall predictive power of EA model was also much higher (R^2^ = 0.807, Figure 2A) than observed for KSVM model (R^2^ = 0.234, Figure 2B). With R^2^ of 0.807 the correlation is considered to be strong [74]. Therefore, the MSR can be successfully predicted using EAs. To check if the numerical relationship is not by chance y-randomization technique was also applied. The method successfully validated the model developed with EA. As can be seen from the results of the y-randomization (Table 3), the real R^2^ was a statistical outlier, as opposed to the y-randomized R^2^ values, with a p value of 0.006, indicating that the relationship between the MSR and descriptors is non-random.

In predicting logMSR kernel support vector machines (KSVM) did not perform satisfactorily (R^2^_val_ = 0.166 ± 0.152, R^2^ = 0.234). Although, KSVM is considered an effective regression tool for small datasets, it still suffers from the curse of dimensionality [75]. Although it handles small datasets better than other regression techniques it still may become useless if the ratio of observations to features is not big enough. The genetic algorithm approach, like the one presented by Eureqa, is much more likely to succeed in case of data sparsely distributed in the feature space. If the amount of the data does not allow to estimate the functional relationship with statistical methods, fitting a surface may still provide a tool for prediction.

Support vector machines are known to be useful in case of small datasets as it takes into consideration the points that support the margin [76]. However, the dataset has to be large enough to provide the sufficient number of points supporting the margin. Otherwise, it is impossible to build the function in kernel space that would reflect the real relationships hidden in the data. If the dataset is unable to provide sufficient number of support vectors then the prediction becomes poor.

In the considered case, we are unable to build a reliable KSVM predictor for the given data, due to its insufficient size.

### 2.3. Sensitivity Analysis

**Figure 3:**
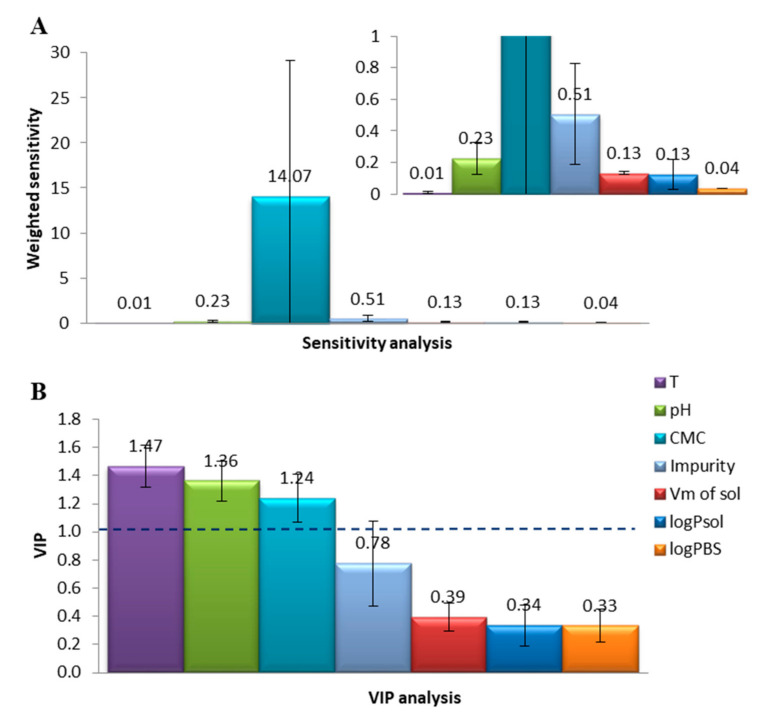
Sensitivity analysis for modelling of logMSR in biosurfactant solutions: (**A**) Average sensitivity towards each of descriptors weighted according to number of appearances, obtained with evolutionary algorithm (EA); (**B**) average variable importance in projection (VIP) analysis performed with PLS regression method. Error bars represent 95% CI. The Eureqa evolutionary algorithm (EA) provides random model equations. The error in the sensitivity of the CMC is due to the large range from 0.78 to 158 as shown in Table S5.

All ten models (given in Table S5) created with EA for the dataset were subjected to the sensitivity analysis [36]. The results of the analysis are shown in Figure 3A,B. As can be seen from Figure 3A, according to EA sensitivity analysis MSR strongly depends on CMC, and the influence of other parameters remains negligible. The importance of CMC was confirmed by PLS analysis (VIP ≥ 1 [77]). Here in turn, also pH and T were found to significantly alter solubilization efficiency (Figure 3B).

The results of sensitivity analysis are in agreement with literature. The dependence of the MSR on CMC is evident from Equation (1), as micellar solubilization requires the presence of micelles. As CMC is sensitive to pH changes, therefore this environmental factor appears to alter the overall solubilization efficiency. Further, temperature may also affect MSR. Just as in case of pH, this happens indirectly—by influencing CMC that already appeared crucial for MSR prediction within both sensitivity analyzes (Figure 3A,B).

Surprisingly, the nature of solubilizate, namely its hydrophobicity/hydrophilicity (expressed here as logP_sol_) seems to have no apparent effect on solubilization. Although literature concerning micellar solubilization indicates that strongly hydrophobic solubilizates (e.g., alkanes) incorporate in the core of micelle, and hydrophilic solubilizates incorporate in palisade layer [78,79], this effect was found negligible for predicting MSR by both VIP and EA sensitivity analyzes (Figure 3A,B).

### 2.4. PCA and Descriptor Overlap

**Figure 4:**
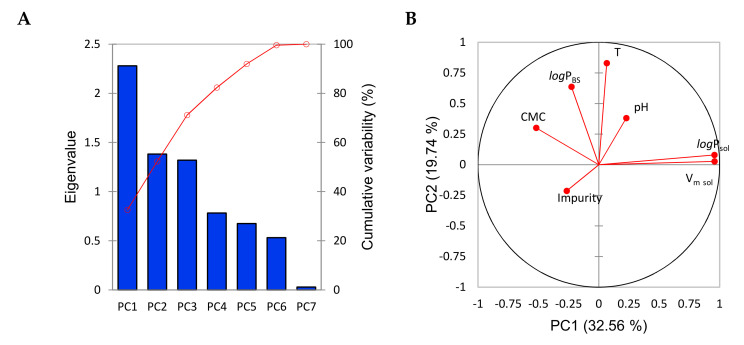
The results of the PCA analysis are shown. (**A**) shows the scree plot of the eigen-values, indicating that the data can be reduced to about six variables. In (**B**), correlation plot of descriptors is presented.

PCA is a technique that projects the data into another feature space in which the arising features are uncorrelated. The data features in the new space are called principal components (PCs). The PCs are ordered due to the decreasing amount of information (Eigenvalue) introduced into the dataset. They are usually presented in the form of a bar chart (Figure 4A). Such knowledge allows to reduce the data dimensionality as the vectors positioned at the last places usually introduce small amounts of information and can be neglected without the significant losses. Apart from dimensionality reduction, PCA can be used to estimate the correlation relationships of the original features. For this purpose projection of the original features onto the plane created by the two most significant PCs is done. The graphical presentation of this is the correlation plot (Figure 4B). In such a plot vectors pointing along the similar lines are correlated. If they point at the same direction the correlation is positive, if opposite the correlation is negative. As can be seen on Figure 4B, both V_m_ and logP of the solubilizate are strongly overlapping, indicating that they provide similar information to the model. The similarity of logP_sol_ and V_m sol_ is clear, as a larger V_m_ sol results in a larger logP_sol_, considering the collected range of the solubilizates. The PCA results are in accordance with the results provided by EA sensitivity analysis. None of 10 EA models delivered used these two descriptors simultaneously (Table S5). Other variables are uncorrelated with any of the other features.

## 3. Materials and Methods 

### 3.1. Materials

Rhamnolipid biocomplex (RBC) was biosynthesized by *Pseudomonas* sp. PS-17 as described previously [36] and is a complex of RLs with an exopolysaccharide, alginate. JBR 425 (lot. No. 040714) in a form of the 25% neutral aqueous solution of dirhamnolipid and monorhamnolipid at 0.97:1 (*w/w*) ratio was kindly provided by the Jeneil Biosurfactant Company (Saukwille, WI, USA). This sample is referred to pure RLs. Sodium alginate (W201502) was purchased in Sigma-Aldrich (Poznań, Poland). Dodecane (99+, Lancaster) was purified from polar additives by passing through a column filled with calcinated Al_2_O_3_ (120 °C, 2 h). Isopropanol (p.a., POCh Gliwice, Poland) was used as a solvent for GC analysis.

### 3.2. Dodecane Solubilization

Rhamnolipid biocomplex (RBC), pure RLs (JBR425) and their mixtures with alginate at 1.3:1 and 1:1 weight ratio (referred to model RBCs) were used as biosurfactant preparations of different purity for the current research. Aqueous solutions of the biosurfactant preparations at the following concentrations 1.0, 2.5, 5.0, 7.5, and 10.0 g/L were prepared by series dilution of 10 g/L stock solution. Ultrapure water (D4700, Barnstead) was used for preparing the solutions. NaOH and HCl were used to set the pH. Aqueous (5 mL) and oil phases (0.5 mL of dodecane) were mixed in 10 mL vials at 1500 rpm for 24 h with a horizontal shaker (IkaVibrax VXR, Ika Technologies, Boutersem, Belgium) at room temperature. Unsolubilized dodecane (excess) was separated from micellar phase by centrifugation at 5000 rpm for 15 min (MPW-350R, MPW Med. Instruments, Warsaw, Poland). Subsequently, clear micellar phase was separated with a syringe through a silicone sealed hole in a bottom of the vial and analyzed for the content of dodecane. All experiments were run in triplicate.

### 3.3. Solubilization Efficiency

Gas chromatography was used for determination of dodecane solubilization efficiency. One milliliter of micellar phase was diluted with 4 mL of 2-propanol. Chrompack CP 9001 with XTI-5 capillary column (30 m × 0.32 mm, 1 μm) was used for GC analysis. The analysis was performed at 180 °C, using a split feeder and FID at 250 °C. The injection volume was 1 μL. An external calibration was used for quantitative analysis.

A simplified equation 1 was used to calculate MSR values. As the solubility of dodecane is negligible, C_CMC_ ~ 0 was assumed. Considering high surfactants concentrations used as compared to CMC, and linearity of C = f(C_surf_) function in the examined range of surfactant concentrations, the MSR equals to the slope (i.e., directional factor *a*) as follows:

(2) $$\mathrm{MSR}\;=\;\frac{C-C_{CMC}}{C_{surf}-CMC}=\frac{C}{C_{surf}}=\frac{\Delta C}{\Delta C_{surf}}=tg\alpha =a$$

where *α* is a slope of *C* = *f*(*C_surf_*) function, and *a* is a directional factor of thereof.

### 3.4. Data Collection

The modelling was performed on a dataset of MSR values collected from literature and experimental data obtained for the purpose of this research. Google Scholar, which provides a high coverage [80], was used for literature data collection. The years 1980–2017 were covered in the search, however the first article was found in 1994. The following keywords were used to search for the MSR data: Biosurfactant, rhamnolipid, monorhamnolipid, dirhamnolipid, sophorolipid, emulsan, lichenisyn, surfactin, solubilization, MSR, and WSR (molar and weight solubilization ratio, respectively). As a result, 21 publications were found, resulting in 44 unique data points. However, only 11 of them provided continuous data needed for modelling, resulting in a dataset with 23 literature data points. Missing data in remaining 16 discontinuous datapoints were filled with Multiple Imputation by Chained Equations (MICE) technique. Finally, the dataset used for modelling consisted of 45 datapoints, out of which six were determined experimentally for the purpose of this research (Table 2 and ESI: MSR Dataset). WSR data were recalculated into MSR value using the Equation (S1). CMC was taken from each paper. Structural descriptors of solubilizate and rhamnolipid (molecular volume V_m_ and logarithm of octanol-water partition coeffiecient logP) were calculated using Molinspiration [81]. If the rhamnolipid was present as a mixture, the weight percent mean was used, as shown in Tables S2 and S3. The state of biosurfactant purification was expressed numerically based on reversed purity scale published before [36] and is presented in Table S4.

### 3.5. MICE

Multiple Imputation by Chained Equations (MICE) was used to extend the dataset (composed initially of 29 data points), and fill the missing data gaps. Here we used an implementation of MICE within XLSTAT (Adinsoft) [82]. Predictive mean matching was used with 14 iterations. MICE methodology was validated as described by Łozińska et al. [77] The normalized mean root squared error (NMRSE) graph is shown in Supplementary Figure S1. MICE was utilized here to show that the model can potentially also be applied to a larger variety of biosurfactants (i.e., alasan, and flavolipids).

### 3.6. Computational Modelling

The dataset was randomly divided into training and validation sets at 8:2 ratio [37,83]. This allows for external validation of the model [84]. In total 10 random training and validation sets were created in order to obtain a truly representative relationship. This methodology has been described in detail by [37,85].

Two methods for constructing models were applied. One was a parametric regression method in which an expression is fitted to the provided data. The expression was constructed by evolutionary algorithm (EA). For this purpose EureqaPro software was used (v1.24.0, build 9367). As the second kernel support vector machine (KSVM) regression was performed. For this purpose, kernlab package in R [86] was used.

In total seven molecular descriptors related to MSR were used to develop a model. Those included physical (CMC) and chemical descriptors (logP of BS and solubilizate, impurity of BS), molecular volumes (V_m sol_) and conditions present in aqueous system (pH, T). The models were searched in a form of the following function logMSR = f(CMC, logP_BS_, (im)purity, logP_sol_, V_m sol_, pH, T).

Detailed descriptions of EA, PLS, and KSVM techniques have previously been provided by us elsewhere [36,77].

### 3.7. EA Generations

**Figure 5:**
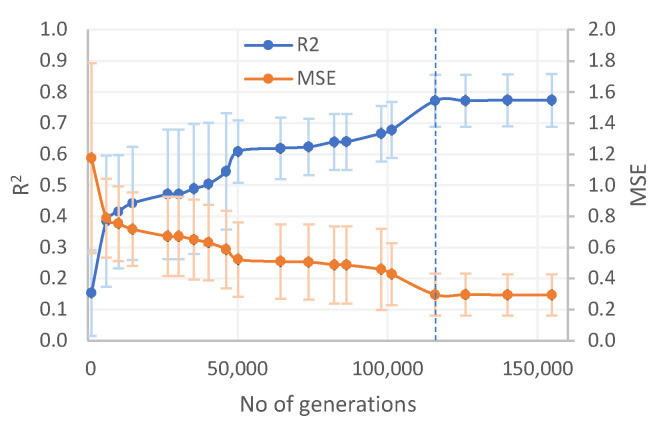
Determination of the modeling endpoint by the analysis of mean R^2^ and mean MSE with SD. The dashed line indicates the endpoint at 115,000 generations. The endpoint was chosen due to the plateau region and lower SD in MSE and R^2^.

Due to the fact, that EureqaPro generates different equations for the same data sets each instance it is run, modelling was repeated ten times to assure high reliability of final model (the methodology for this was discussed further in our previous publication [36]). Based on those ten measurements average R^2^ and average mean squared error (MSE) were calculated to determine the end point of modelling (Figure 5).

### 3.8. Model Validation—Y-Randomization

Y-randomization was performed to exclude an accidental relationship between the descriptors, and the dependent variable (MSR). In this case, 10 randomizations of the MSR were conducted. The descriptors were left unchanged. Each set was modelled separately using the EA as described before. The resulting R^2^ was compared to the non-randomized R^2^ of the original set, and the Grubbs outlier test was used to determine the *p*-value of the outlier.

### 3.9. SVM Regression Methodology

In order to perform KSVM regression each of the data features was rescaled to the range of values varying from −1 to 1. A Gaussian kernel was chosen as this is usually the best one for non-linear data [87]. The set of optimal hyper-parameters was determined using the grid search with leave one out cross validation. The error margin tolerance was set to ε = 0.01.

### 3.10. Sensitivity for EA and KSVM

The sensitivity of the model towards descriptors was analyzed only in case of the EA outcome, as KSVM did not provide satisfactory results. Such sensitivity analysis was performed with two methods, namely using EA sensitivity analysis [36] and partial least squares variable importance of projection (PLS VIP) analysis [77,88]. The XLSTAT implementation of PLS regression was used [82,89]. For cross validation, the jackknife leave one out method was used [90]. The variables importance for the projection VIPs were determined according to [91]. PCA was also conducted within XLSTAT.

**Figure 6:**
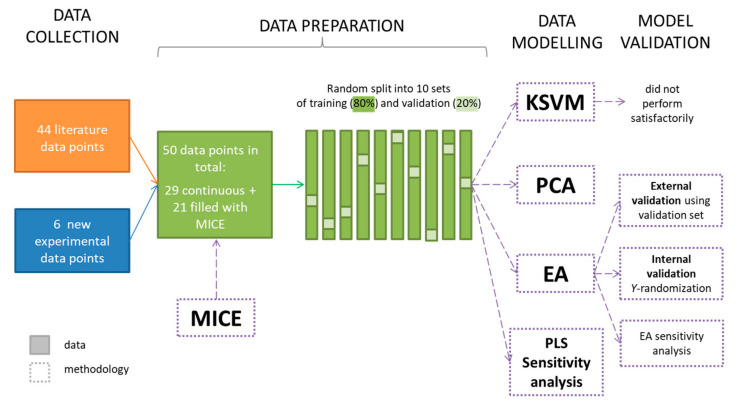
Schematic representation of the procedure used to create the models. EA — evolutionary algorithm; KSVM—kernel support vector machine; MICE—multiple implementation by chain equations; PCA—principal component analysis; PLS—partial least squares.

An overall schematic of the research procedure is shown in Figure 6.

## 4. Conclusions

To our knowledge the prediction of solubilization efficiency (MSR) in RLs solutions was described for the first time. We have shown that MSR can be successfully predicted with EA (R^2^_val_ = 0.773 ± 0.052). As opposed to HLD concept [48,49], vast majority of descriptors for our MSR approach can be easily calculated with a software widely available online. Therefore, the experimental effort is minimized.

Additionally, we have ranked the parameters influencing the MSR upon their significance. This in turn may enhance the ease of designing experimental work with rhamnolipids, as the most crucial parameters for rhamnolipid solubilizing properties were determined and systematized.

The model will allow biosurfactants to be designed for specific applications, which will enhance the use of these green surface active molecules. What is more, we aimed at changing the approach of surfactant application. Currently, research is often aimed to determine the best application for a surfactant that was either selected or bio/synthesized for the purpose of research [92,93,94,95]. However, here we propose to determine the optimal surfactant a priori for the application of interest, e.g., to pre-select best rhamnolipid biosurfactant for removing oil spill of known composition. We propose here the following hypothetical scenario, where we assume that on one spring day (T = 20 °C) a *p*-xylene (logP = 2.83, V_m_ = 117.17 Å^3^ [81]) tank truck is damaged due to road incident and the chemical spills over the road and neighboring acidic ground (pH 5), which raises an urgent need for ground remediation. The MSR calculator that we provide (ESI MSR calculator) allows for quick selection of a biosurfactant that can be used, indicating the optimal biosurfactant will be found among alasan (calculated MSR = 19.0), monoRL mixture (calculated MSR = 7.3), or mixture of JBR425 and alginate 1:1 (calculated MSR = 2.34). Combining our new approach with the required parameters of biosynthesis would result in “a recipe for rhamnolipid” for a targeted application, which we are currently investigating further. The MSR model is limited by range of parameters as defined by the applicability domain that comprise the model. It is feasible, for example, that biosurfactants can be developed that have larger or smaller MSR values in the future. The model should then be updated.

## Figures and Tables

**Table 1:** Exemplary results of selective solubilization of components from matrices of biological or environmental significance with an application of different surfactants. Shown are compounds selectively solubilized over other components of the matrix, thereby highlighting the need for a targeted molar solubilization ratio (MSR).

Process	Surfactant	Matrix	Compound Selectively Solubilized	Ref.
Separation of proteins with similar, low molecular size from their ternary mixture	*Anionic surfactant:* Aerosol OT/AOT (sodium di-2-ethylhexyl sulfosuccinate)	Ternary mixture of LMW proteins (ribonuclease-a, cytochrome-c and lysozyme)	Ribonuclease-a or cytochrome-c or lysozyme	[39]
Selective solubilization	*Nonionic surfactants:* Brij 30, C_12_E_4_Brij 35, C_12_E_23_ Tergitol NP10, C_9_PE_10.5_ Triton X-100, C_8_PE_9.5_Tween 80, C_18_S_6_E_20_	Naphthalene, hexadecane and/or phenantrene	Naphtalene or phenantrene	[40]
Selective solubilization	*Cationic surfactants:* Cetylpyridinium chloride, C_16_PyrCl Dodecyl ammonium chloride, C_12_NH_3_Cl Dodecyl trimethyl ammonium chloride, N[C12,C,C,C]Cl *Anionic surfactants:* Sodium dodecyl sulfate, C_12_SO_4_Na Sodium diamyl sulfosuccinate (Aerosol AY)	Benzene-hexane mixtures	Benzene	[41]
Selective solubilization of PAHs from NAPL	*Nonionic surfactants:* Brij 30, C_12_E_4_ Brij 35, C_12_E_23_ Tergitol NP10, C_9_PE_10.5_ Triton X-100, C_8_PE_9.5_Tween 80, C_18_S_6_E_20_	Ternary mixtures of naphtalene, phenantrene and hexadecane	Phenantrene ^1^	[42]
Competitive solubilization of naphthalene and pyrene	*Nonionic surfactants:* Brij30, C_12_E_4_ Brij56, C_16_E_10_ *Cationic surfactants:* Dodecylethyldimethyl ammonium bromide, N[C12,C2,C,C]Br Cethyltrimethyl ammonium chloride, N[C16,C,C,C]Br *Anionic surfactant:* Sodium dodecyl sulfate, C_12_SO_4_Na	Binary mixtures of pyrene and naphthalene	Pyrene	[38]
Selective solubilization of glycerolipid and glycoprotein species from isolated human erythrocyte membranes	*Nonionic surfactant:* Triton X-100	Isolated human erythrocyte membranes	Glycolipid, glycerolipid	[43]

^1^ or reverse, depending on the type of surfactant used in experiment; E—oxyethylene group; PE—oxypropylene group; types of surfactants were marked in italic.

**Table 2:** Range and diversity of different parameters used for creation of model to predict MSR of BS in aqueous system. These represent the bounding box applicability domain according to Sahigara et al. [52].

Parameter	Impurity	logP_BS_	CMC [mg/L]	logP_sol_	V_m_ sol. [Å^3^]	pH	T [°C]	MSR
Min. value	0	4.51	0.0010	2.69	94.19	4	20	1.35 × 10^−5^
Max. value	2	5.21	700.00	9.00	314.59	9	30	82.10
Mean	0.73	4.88	101.98	5.80	207.04	6.76	24.0	4.97
SD	0.80	0.22	118.20	1.99	59.42	0.78	2.7	16.06

**Table 3:** Parameters obtained for ten models, used to obtain formula able to predict logMSR, after 115,000 generations. Average R^2^_val_ and average MSE_val_ were calculated for ten validation sets. Also shown are results of the *y*-randomization, indicating that the relationships are not due to random chance.

Parameter	Chemometric Tool	
	EA	KSVM
External validation:		
R^2^_val_ ± CI	0.773 ± 0.052	0.166 ± 0.152
MSE_val_ ± CI	0.296 ± 0.084	1.176 ± 0.483
Y-randomization:		
G	2.613	-
G (Critical value)	2.355	-
*p*-value	0.006	-

## Data Availability

The data presented in this study are available in electronic supplementary information [ESI, ESI MSR dataset with MICE, ESI MSR calculator].

## Supplementary Materials

The following are available online, complete dataset of MSRs used for prediction, EA equations, and MSR calculator.

## Sample Availability

Samples of the RBC are available from the authors.

## Abbreviations

CI, confidence interval; CMC, critical micellar concentration; EA, evolutionary algorithm; KSVM, kernel support vector machines; logP, logarithm of octanol-water partition coefficient; MSE, mean square error; MSR, molar solubilization ratio; NRMSE, normalized root mean squared error; PC, principal component; PCA, principle component analysis; QSPR, quantitative structure–property relationship; RBC, rhamnolipid biocomplex with alginate; SD, standard deviation; V_m_, molecular volume.
