# Supplementary material for: Towards Rational Biosurfactant Design—Predicting Solubilization in Rhamnolipid Solutions
Source: Molecules. 2021 Jan 20;26(3):534. doi: 10.3390/molecules26030534 (PMC7864340; doi:10.3390/molecules26030534)
Supplement: Supplementary file 1 [file molecules-26-00534-s001.zip › molecules-1007862-supplementary material.pdf]

molecules

MDPI

Supplementary Material

Towards Rational Biosurfactant Design—Predicting Solubilization in Rhamnolipid Solutions

Ilona E. Kłosowska-Chomiczewska [1,*], Adrianna Kotewicz-Siudowska [1], Wojciech Artichowicz [2], Adam Macierzanka [1], Agnieszka Głowacz-Różyńska [1], Patrycja Szumała [1], Krystyna Mędrzycka [1], Elżbieta Hallmann [1], Elena Karpenko [3] and Christian Jungnickel [1]

[1] [1] Department of Colloid and Lipid Science, Faculty of Chemistry, Gdańsk University of Technology, Narutowicza St. 11/12, 80-233 Gdańsk, Poland; adriannakotewicz@gmail.com (A.K.-S.); adamacie@pg.edu.pl (A.M.); agnglowa@pg.gda.pl (A.G.-R.); patszuma@pg.edu.pl (P.S.); kbm@pg.edu.pl (K.M.); hallmann.elzbieta@gmail.com (E.H.); cjungnickel@googlemail.com (C.J.)

[2] Department of Hydraulic Engineering, Faculty of Civil and Environmental Engineering, Gdańsk University of Technology, Narutowicza St. 11/12, 80-233 Gdańsk, Poland; wojartic@pg.edu.pl

[3] Department of Physical Chemistry of Fossil Fuels InPOCC, National Academy of Sciences of Ukraine, 3a Naukova St., Lviv, 79053, Ukraine; e.v.karpenko@gmail.com

* Correspondence: ilochomi@pg.edu.pl; Tel.: +48-58-347-11-51

Table S1. Comparison of CMC values of RLs with selected synthetic surfactants.

Surfactant	CMC, g/L	CMC, mM	Surfactant Type	Ref.
Rhamnolipidbiocomplex	0.0621 at pH 7	0.078	anionic	[1]
Rhamnolipidbiocomplex	0.0415 at pH 9	0.052	anionic	[1]
Rhamnolipid JBR 425	0.0367 at pH 7	0.064	anionic	[1]
Rhamnolipid JBR 425	0.0439 at pH 9	0.076	anionic	[1]
Rhamnolipid JBR 425	0.07	0.1215	anionic	[2]
Sodiumdodecylsulphate (SDS)	2.31	8	anionic	[3]
Sodium soap $C_{12-18}Na$, average molecular formula $C_{13.4}Na$	0.423	1.75	anionic	[4]
Sodium dioctyl sulfosuccinate (Aerosol OT or AOT)	1.21	2.72	anionic	[5]
Sodiumperfluorooctanesulfonate (PFOS)	4.84	8.9	anionic	[6]
Sodiumperfluorooctanoate (PFOA)	16.57	38	anionic	[6]
Cetylpirydynium chloride	0.326	0.96	cationic	[5]
Ethoxylated alcohol (Rokanol NL6), $C_{9-11}E_6$	0.12	0.2857	nonionic	[2]
AlkypolyglucosideC_8–$C_{10}G_{1.6}$, average molecular formula $C_{8.6}G_{1.6}$	0.636*	1.63	nonionic	[4]
Ethoxylated alcohol $C_{12-14}E_7$, average molecular formula $C_{13}E_7$	0.132*	0.26	nonionic	[4]
Polyethylene glycol sorbitanmonooleate (Tween 80)	0.016	0.012	nonionic	[7]
Polyethylene glycol sorbitanmonolaurate (Tween 20)	0.072	0.059	nonionic	[7]

* Molecular masses of compounds were calculated as described by Rodriguez et al. in [8].

Equation S1. The main difference between MSR and WSR is the final unit (mol/L and mg/L, respectively) that is why for MSR calculation WSR values were just multiplied by the ratio of molar mass of BS used in research and molar mass of solubilizate:

$$\text{MSR}= WSR \cdot \left(\frac{molar\ mass\ of\ BS}{molar\ mass\ of\ solubilizate}\right) \quad \text{S1}$$

Table S2. Molar mass of each possible combination of mono and di RL.

Mono and Di RL Structures	Molar Mass
RhaC10:C8	476
RhaC10:C10:1*	502
RhaC10:C10	504
RhaC10:C12:1*	530
RhaC10:C12	532
RhaRhaC8C10	620
RhaRhaC10C8	620
RhaRhaC10C10	648
RhaRhaC10C12:1*	674
RhaRhaC12:1C10*	674
RhaRhaC10C12	676
RhaRhaC12C10	676

*one double bond present in the structure.

Table S3. Assumptions used for calculation of logP of BS taken from Molinspiration [9]. For an exemplary mixture of 60 % *w/v* monorhamnolipid (C26H48O9, MW = 504) and 40 % *w/v* dirhamnolipid (C32H58O13, MW = 650) the mean logP equals to 4.93.

RL type	logP	V_m
Mono RL	5.21	501.49
Di RL	4.51	625.35

Table S4. Five classes of purity of BS estimated by scientists in the literature [1] were used in order to calculate impurity with the following formula: impurity = 5 − purity class of used BS.

Purity class	Description
1	culture broth
2	supernatant (cell free culture broth)
3	acidic precipitate (separated from supernatant via acidic precipitation)
4	solvent extracted RL
5	single RL or mixtures of RL separated via e.g., column chromatography

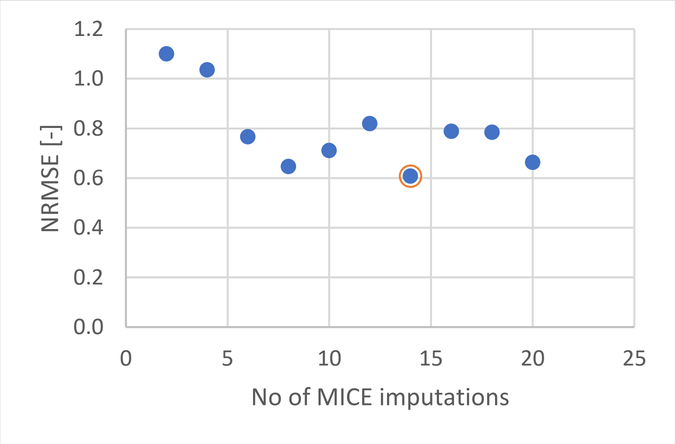

Figure S1. Influence of imputations number on normalize mean root squared error (NRMSE) of filling missing data with MICE technique. Optimal number of imputations was chosen (14) as the one providing smallest NRMSE.

Table S5. Data generated as a sensitivity report using EureqaPro evolutionary algorithm at 115,000 generations, based on 10 modeled sets of collected data.

Set No.	Equation	R^2	Variable	Sensitivity	% positive	Positive Magnitude	% Negative	Negative Magnitude
1	$logMSR = 0.0549013360444942 \times T + T/(CMC - 189.159775924084) + 0.84003033066106 \times CMC/(150.387519123862 - logP_{sol} \times CMC) - 1.45795564997864$	0.898	CMC	13.763	56	18.408	44	7.8789
			$logP_{sol}$	3.1013	64	4.1426	36	1.227
			T	0.17639	100	0.17639	0	0
			-	-	-	-	-	-
2	$logMSR = 0.0980088330223142 \times T + 19.7433722499227/(CMC - 191.277327609993) + 8.7307980450418 \times Impurity/(CMC - 21.0843762671156) - 2.92039790020091$	0.932	CMC	155.06	6	1053.5	94	98.908
			Impurity	1.5282	0	0	100	1.5282
			T	0.29151	100	0.29151	0	0
			-	-	-	-	-	-
3	$logMSR = T/(CMC - 188.585866912513) + (-21.797302639218)/(CMC^2 - 319.231653068273) + (0.914065837624662 \times CMC^3 - 146.624787660455)/(82790.6730149424 \times CMC - 1476.81156395048 \times CMC^2) - 0.144427838055886$	0.692	CMC	42.395	65	62.025	35	6.4081
			T	0.0033173	0	0	100	0.0033173
			-	-	-	-	-	-
			-	-	-	-	-	-
4	$logMSR = 0.0515592636477317 \times CMC + (-6.49185514010058)/(4.1859811938078 \times CMC - 237.273069939631) + (-0.15749508416671) \times CMC/(18.7287681388235 - CMC) -$	0.764	CMC	11.77	79	11.593	21	12.454
			pH	0.6693	0	0	100	0.6693
			-	-	-	-	-	-
			-	-	-	-	-	-

	1.15999405611935 − 0.00640548553892461 × pH × CMC − 9.15198712813605 × 10^{-6} × CMC^2							
5	logMSR = 41.5310569032383/(435.191324875112 + 48.0977907300403 × Impurity × $logP_{BS}$ − 0.118708305249961 × $V_{m\ sol}$ × T) − 0.236384375536414	0.801	Impurity	7.2818	50	4.5358	50	10.028
			Vm_{sol}	1.7037	75	1.7378	25	1.6014
			$logP_{BS}$	1.0667	4	12.379	96	0.57489
			T	0.42569	67	0.61747	33	0.04214
6	logMSR = 0.187249061212849 × Impurity + 0.100745907422626 × T + 0.000378569818964285 × CMC^2 + (−48.9853072239654) × Impurity/(67.4618302151054 − 0.154096608070562 × CMC^2) − 3.14741488870321 − 5.4098924966292 × 10^{-5} × pH × CMC^2	0.757	CMC	158.79	38	252.82	62	100.58
			Impurity	1.9751	100	1.9751	0	0
			pH	1.3237	0	0	100	1.3237
			T	0.31692	100	0.31692	0	0
7	logMSR = 21.9623107647302/(CMC − 188.6496) − 0.324146181651744	0.716	CMC	1.8889	3	27.253	97	1.1203
			-	-	-	-	-	-
			-	-	-	-	-	-
			-	-	-	-	-	-
8	logMSR = T/(CMC − 188.916558292979) − 0.48089142702189	0.704	CMC	1.8443	3	26.701	97	1.091
			T	0.0045123	0	0	100	0.0045123
			-	-	-	-	-	-
			-	-	-	-	-	-

9	logMSR = 0.0605457488939214 × T + 17.0895137018274/(CMC − 190.23740116534) + 2267.32189621285/(30404.9129504339 + 16074.112197658 × Impurity − 189.254674927053 × $V_{m\ sol}$) − 1.61641580966156	0.766	Vm_{sol}	1.9673	75	2.0927	25%	1.5912
			Impurity	1.533	25	1.4508	75%	1.5603
			CMC	0.77689	3	11.452	97%	0.45339
			T	0.17164	100	0.17164	0%	0
10	logMSR = ($logP_{sol}$ + 1.3122084034934 × T − 21.7058791564727)/pH + (6.62847525310523 × CMC − 185.846282207051)/(1.61668654130701 × Impurity × pH × CMC − 41.6569686153301 × pH) − 2.68009390108419	0.693	CMC	7.7384	12	20.019	88	6.101
			Impurity	1.8856	63	1.6851	38	2.2197
			T	0.61693	100	0.61693	0	0
			$logP_{sol}$	0.41258	100	0.41258	0	0
			pH	0.38095	0	0	100	0.38095

References

1. Kłosowska-Chomiczewska, I.; Mędrzycka, K.; Hallmann, E.; Karpenko, E.; Pokynbroda, T.; Macierzanka, A.; Jungnickel, C. Rhamnolipid CMC prediction. *J. Colloid Interface Sci.* **2017**, *488*, 10-19, doi:10.1016/j.jcis.2016.10.055.
2. Medrzycka, K.; Hallmann, E.; Pastewski, S. Evaluation of surfactant and biosurfactant mixture usefulness in oil removal from soil, based on physicochemical studies and flushing experiments. *Environ. Prot. Eng* **2009**, *35*, 191-205.
3. Özdemir, G.; Malayoglu, U. Wetting characteristics of aqueous rhamnolipids solutions. *Colloids Surf. B* **2004**, *39*, 1-7, doi:10.1016/j.colsurfb.2004.08.006.
4. Szumała, P.; Mówińska, A. Perfectly wetting mixtures of surfactants from renewable resources: the interaction and synergistic effects on adsorption and micellization. *J. Surfactants Deterg.* **2016**, *19*, 437-445, doi:10.1007/s11743-016-1793-z.
5. Chatterjee, A.; Moulik, S.; Sanyal, S.; Mishra, B.; Puri, P. Thermodynamics of micelle formation of ionic surfactants: a critical assessment for sodium dodecyl sulfate, cetyl pyridinium chloride and dioctyl sulfosuccinate (Na salt) by microcalorimetric, conductometric, and tensiometric measurements. *J. Phys. Chem. B* **2001**, *105*, 12823-12831, doi:10.1021/jp0123029.
6. Banerjee, S.; Schmidt, J.; Talmon, Y.; Hori, H.; Asai, T.; Ameduri, B. A degradable fluorinated surfactant for emulsion polymerization of vinylidene fluoride. *Chem. Comm.* **2018**, 10.1039/C8CC05290E, doi:10.1039/C8CC05290E.
7. Chou, D.K.; Krishnamurthy, R.; Randolph, T.W.; Carpenter, J.F.; Manning, M.C. Effects of Tween 20® and Tween 80® on the stability of Albutropin during agitation. *J. Pharm. Sci.* **2005**, *94*, 1368-1381, doi:10.1002/jps.20365.
8. Rodriguez, V.B.; Alameda, E.J.; Requena, A.R.; López, A.G.; Bailón-Moreno, R.; Aranda, M.C. Determination of average molecular weight of commercial surfactants: alkylpolyglucosides and fatty alcohol ethoxylates. *J. Surfactants Deterg.* **2005**, *8*, 341-346, doi:10.1007/s11743-005-0366-y.
9. Chemoinformatics, M. Mol inspiration. Brastislava, Slovak Republic, 2014.
